# miR-150 Promotes Human Breast Cancer Growth and Malignant Behavior by Targeting the Pro-Apoptotic Purinergic P2X_7_ Receptor

**DOI:** 10.1371/journal.pone.0080707

**Published:** 2013-12-02

**Authors:** Songyin Huang, Yongsong Chen, Wei Wu, Nengyong Ouyang, Jianing Chen, Hongyu Li, Xiaoqiang Liu, Fengxi Su, Ling Lin, Yandan Yao

**Affiliations:** 1 Department of Laboratory, Sun Yat-Sen Memorial Hospital, Sun Yat-Sen University, Guangzhou, Guangdong, China; 2 Department of Endocrinology, The First Affiliated Hospital, Shantou University Medical College, Shantou, Guangdong, China; 3 Breast Tumor Center, Sun Yat-Sen Memorial Hospital, Sun Yat-Sen University, Guangzhou, Guangdong, China; 4 Department of Gynaecology and Obstetrics, Sun Yat-Sen Memorial Hospital, Sun Yat-Sen University, Guangzhou, Guangdong, China; 5 Department of Rheumatology, The First Affiliated Hospital, Shantou University Medical College, Shantou, Guangdong, China; University of Barcelona, Spain

## Abstract

The P2X_7_ receptor regulates cell growth through mediation of apoptosis. Low level expression of P2X_7_ has been linked to cancer development because tumor cells harboring a defective P2X_7_ mechanism can escape P2X_7_ pro-apoptotic control. microRNAs (miRNAs) function as negative regulators of post-transcriptional gene expression, playing major roles in cellular differentiation, proliferation, and metastasis. In this study, we found that miR-150 was over-expressed in breast cancer cell lines and tissues. In these breast cancer cell lines, blocking the action of miR-150 with inhibitors leads to cell death, while ectopic expression of the miR-150 results in increased cell proliferation. We deploy a microRNA sponge strategy to inhibit miR-150 in *vitro*, and the result demonstrates that the 3′-untranslated region (3′UTR) of P2X_7_ receptor contains a highly conserved miR-150-binding motif and its direct interaction with miR-150 down-regulates endogenous P2X_7_ protein levels. Furthermore, our findings demonstrate that miR-150 over-expression promotes growth, clonogenicity and reduces apoptosis in breast cancer cells. Meanwhile, these findings can be decapitated in nude mice with breast cancer xenografts. Finally, these observations strengthen our working hypothesis that up-regulation of miR-150 in breast cancer is inversely associated with P2X_7_ receptor expression level. Together, these findings establish miR-150 as a novel regulator of P2X_7_ and a potential therapeutic target for breast cancer.

## Introduction

Breast cancer is the most common cancer afflicting women around the world [1], and distal metastasis of highly invasive breast cancer cells is the major cause of death in these women. Recently, the classical categories of oncogenes and tumor suppression genes have been expanded to include a new family of RNAs known as microRNAs (miRNAs), which may regulate a large number of protein-coding genes, including tumor-related genes. miRNAs are a class of endogenous 22–24 nt non-coding single-stranded RNA molecules that regulate gene expression post-transcriptionally. miRNAs can affect multiple cell processes including proliferation, apoptosis, differentiation, angiogenesis, and development [2], [3]. Not only do they inhibit translation of their target genes, they also degrade the target mRNAs through recognition of imperfect complementary sites, usually located in the 3′-untranslated regions (3′UTR) of the target mRNAs, endowing miRNAs with the capacity to regulate numerous biological processes. Loss or gain of function of specific miRNAs contributes to tumorigenesis and cancer progression. miR-150, a hematopoietic cell-specific miRNA, was shown to affect B-cell differentiation and development [4]. Most of the studies have indicated that miR-150 is significantly over-expressed in multiple kinds of cancers, including malignant lymphoma, and gastric, lung, endometrial, and pancreatic cancers [5], [6], [7], [8], and displays various effects on cellular proliferation, differentiation, apoptosis, migration, and invasion. In recent years, important advances have been made in the knowledge of functions and mechanisms of miR-150 in various human tumors, and several important targets, such as c-myb [4], EGR2 [6], MUC4 [7], P2X_7_ [8], AKT2 [9] and CXCR4 [10] have been identified and experimentally tested for their functional participation in the disease process. However, little is known about the expression and biological role of miR-150 in breast cancer.

The receptor P2X_7_ is the main physiological pro-apoptotic mechanism in epithelia *in vivo*. P2X_7_ receptor is a glycosylated G-coupled-membrane-bound receptor protein [10], and its natural ligand is ATP [11]. Binding of P2X_7_ receptor by ATP can stimulate various signaling pathways including the TNF-a, TRAIL, p38, JNK/stress activated protein kinase (SAPK) and NF-κB cascades, which can induce proliferation, differentiation and apoptosis [12], [13]. Early studies reported on the effect of P2X_7_ receptor in inflammation [14]. Recent studies focused on the effects of P2X_7_ receptor activation in tumor cells. In epithelia derived from the ectoderm, decreased levels of P2X_7_ receptor in the urogenital sinus and the distal paramesonephric duct are associated with cancer development [15]. P2X_7_ receptor expression can be modulated by factors that regulate transcription, post-translational modification, and glycosylation of the receptor [16], [17], [18]. These effects regulate the growth and proliferation of epithelial cells, and possibly control the development of epithelial cancers *in vivo*. P2X_7_ transcription may be regulated by miR-150 in uterine epithelial cells [8].

In the present study, we have investigated the role of miR-150 in the regulation of P2X_7_ receptor expression in breast cancer cells. Our findings demonstrated that the 3′UTR of P2X_7_ receptor contains a putative binding site for miR-150, which is highly conserved across several mammalian species. Furthermore, we experimentally showed that miR-150 directly targets the 3′UTR of P2X_7_ to suppress its expression. miR-150 stimulates a decrease in P2X_7_ mRNA steady-state levels by targeting instability sites within the 3′UTR of the P2X_7_ gene. These data suggested that the reduced expression of P2X_7_ in cancer epithelial cells is the result of high steady-state levels of miR-150 in cancer cells, which activate the instability domains and decrease P2X_7_ mRNA levels, possibly by inducing degradation of the transcript. Our findings also demonstrated that miR-150 over-expression leads to induced growth, clonogenicity and reduced apoptosis in breast cancer cells. Finally, our data revealed a discordant expression of P2X_7_ receptor at the transcript and protein levels, which is inversely associated with miR-150 expression in malignant clinical specimens. Altogether, our study characterized a novel microRNA-mediated mechanism of P2X_7_ regulation and suggests tumor facilitative actions of miR-150 in breast cancer cells.

## Results

### miR-150 is over-expressed in breast carcinomas tissues and cell lines

**Figure 1:**
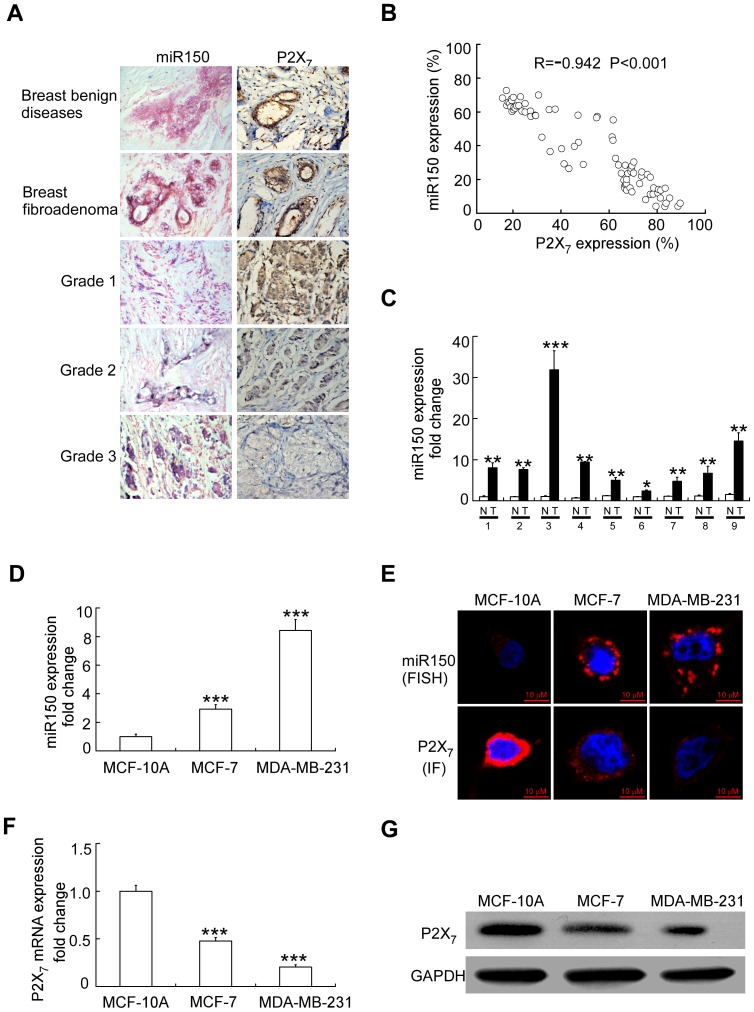
miR-150 levels correlate inversely with P2X_7_ in breast carcinomas and breast cancer cell lines. (A) Representative microscopic images (×400) of in situ hybridization (ISH) for miR-150 and immunohistochemistry for P2X_7_ receptor in breast tumors. (B) The correlation of miR-150 with P2X_7_ receptor protein expression in breast cancer. Pearson correlation coefficients (R) and P-values (p) are indicated. (C) Relative expression of miR-150 (normalized to U6) was detected by using a qRT-PCR in breast tumor tissue sample and matched adjacent non-tumor tissue sample. * p<0.01; ** p<0.01; *** p<0.001, Student's t-test for tumor compared to matched adjacent non-tumor tissue. T, tumor; N, matched adjacent non-tumor tissue. (D) Relative expression of miR-150 (normalized to U6) are using a qRT-PCR in the corresponding breast cancer cell lines (MCF-7 or MDA-MB-231) and human mammary epithelial cell lines (MCF-10A). ***; p<0.001, Student's t-test for MCF-7 or MDA-MB-231 cells compared to MCF-10A cells or MDA-MB-231 cells compared to MCF-7 cells. (E) Microscopic images (×400) of relative expression of miR-150 (FISH) and P2X_7_ receptor (immunofluorescence, IF) in breast cancer cell lines. (F) qRT-PCR data of P2X_7_ mRNA (normalized to GAPDH mRNA) in breast cancer cell lines. ***; p<0.001, Student's t-test for MCF-7 or MDA-MB-231 cells compared to MCF-10A cells or MDA-MB-231 cells compared to MCF-7 cells (G) Western-blotting analysis of the P2X_7_ receptor expression in breast cancer cell lines. Data are the mean of three determinations and shown is representative of three experiments that gave similar results.

*In situ* hybridization was done in 80 paired-samples of breast tumor versus matched adjacent and/or distant non-tumor tissues to analyze differential expression of mature miRNAs. Expression levels of miR-150 in breast cancer tissues were much higher than those in non-tumor tissues (Figure 1A–1C; P<0.05, P<0.01 or P<0.001). Expression levels of miR-150 were closely associated with the degree of malignancy of tumors. Tumors with high malignancy expressed high levels of miR-150, suggesting that miR-150 up-regulation was associated with tumor progression (Figure 1A, Table S1, P<0.01). We also examined the miR-150 expression in breast cancer cell lines MCF-7 and MDA-MB-231 along with the non-malignant breast epithelial cell MCF-10A. The levels of miR-150 were specifically enhanced in human breast cancer cells when compared to normal human mammary epithelial cells MCF-10A (Figure 1D-1E). The expression level of miR-150 was different between MDA-MB-231 (a high-aggressive breast cancer cell line) and MCF-7 (a low-aggressive breast cancer cell line). The high-aggressive cells showed high expression levels of miR-150 while the opposite was true in the low-aggressive cells (Figure. 1D; P<0.001). These data confirmed previous report that more abundant expression of miR-150 in cancer than in normal cells [8], [19]. A previous report reveals that miR-150 levels are lower in both ER^+^ and triple-negative breast tumor specimens compared to adjacent normal epithelium, with triple-negative tumors having the lowest expression [20]. It is tempting to hazard a suggestion that miR-150 may have potential diagnostic or prognostic significance.

To further confirm the role of miR-150 during breast cancer progression, we determined the expression of miR-150 in fresh tumor specimen and adjacent normal breast tissue from 9 patients by using quantitative reverse transcription-PCR. We observed that miR-150 expression was significantly increased in breast cancer tissue compared with adjacent normal breast tissue (Figure 1A).

These data suggested that miR-150 up-regulation is correlated with tumor progression and may play a role in the progression of breast cancers.

P2X_7_, a key factor of apoptosis in epithelial tissues, was also shown as a target of miR-150 [8]. P2X_7_ receptor expression was decreased in pre-cancerous epithelial tissues and cancerous epithelial tissues [21], these findings are biologically and clinically important because defective apoptosis may lead to cancer [22], [23] and the decreased cellular expression of P2X_7_ could be causally related to the development and progression of breast cancers. We examined protein expression of the above genes in paraffin sections of breast cancer samples using immunohistochemistry. In all cases, P2X_7_ immunostaining tissues were reduced in breast cancer compared with adjacent normal breast tissue (Figure 1A). Figure 1F shows qPCR data of P2X_7_ mRNA in human breast epithelial cells and breast cancer cells. When normalized to GAPDH mRNA, P2X_7_ mRNA in breast cancer cells was lower than that in human breast epithelial cells (MCF-10A). Immunofluorescence and western-blotting analysis showed that the P2X_7_ receptor expression in breast cancer cell lines was also lower compared to MCF-10A (Figure 1E, 1G and S1). These data confirmed that P2X_7_ protein and mRNA levels are lower in breast cancer cells compared with human breast epithelial cells [24]. In addition, staining of P2X_7_ receptor in breast cancer tissues was inversely correlated with miR-150 expression (Figure 1A-1B, Table S1; P<0.001). These observations supported previous findings that P2X_7_ is one of target genes silenced by miR-150 [8].

### Down-regulation of miR-150 inhibits breast cancer cell proliferation and induces apoptosis *in vitro*

**Figure 2:**
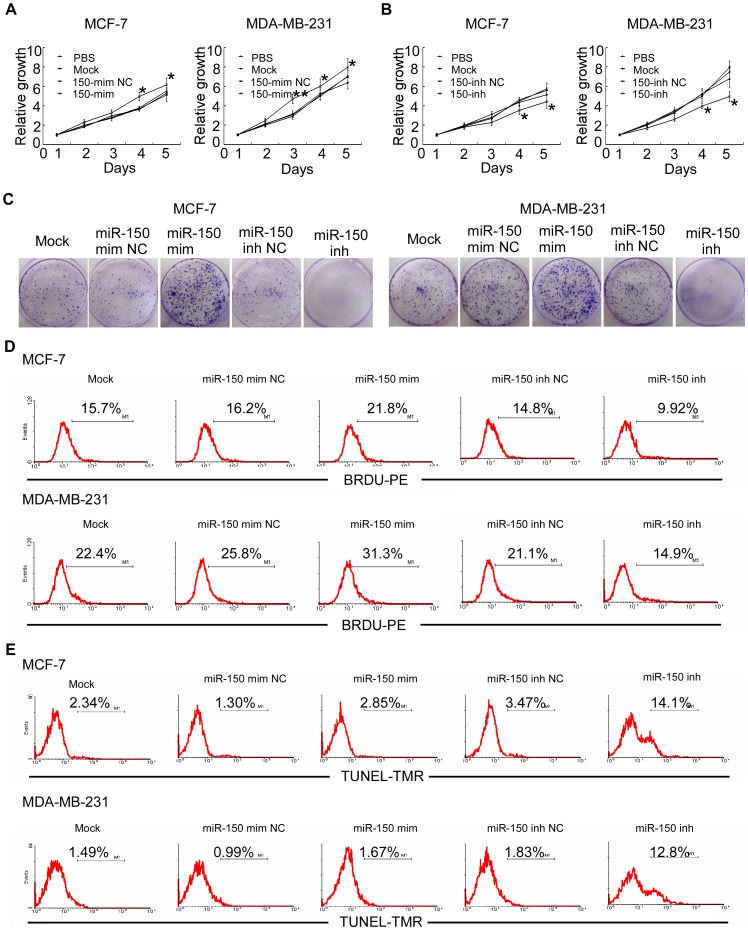
miR-150 promotes the cell growth and inhibits the cell apoptosis of breast cancer cells. (A–B) Proliferation curves of breast cancer cells transfected with miR-150 mimics (150- mim), miR-150 inhibitors (150-inh) and matched negative control (NC) (150- mim NC or 150-inh NC). Cell survival was determined by MTT assay. * p<0.01, One-way ANOVA compared to matched NC, Mock or PBS. (C) Anchorage-independent growth as determined by soft agar colony formation assay. (D) Percentage of BrdU^+^ cells determined by flow cytometric analysis of BrdU immunostaining and representative flow cytometric histograms of BrdU immunostaining in MCF-7 or MDA-MB-231 cells transfected with miR-150 mimics, inhibitors and matched negative control. (E) Cell apoptosis of MCF-7 or MDA-MB-231 cells was performed after transfected with miR-150 mimics, inhibitors and matched NC. Data are the mean of three determinations and shown is representative of three experiments that gave similar results.

To corroborate the function of miR-150 during tumorigenesis, breast cancer cells lines, MCF-7 and MDA-MB-231, were transfected with miR-150 mimics or miR-150 inhibitors. MTT and colony formation assays were performed to examine the effects of miR-150 on *in vitro* cell growth. Our data demonstrated that relative cell growth was significantly facilitated in miR-150 mimics transfected MCF-7 (∼41.2%) or MDA-MB-231 (∼58.9%) cells on day 5 compared with their respective controls (miR-150 mimics NC or inhibitor NC transfected cells) (Figure 2A). As expected, cells receiving antagomirs displayed the opposite effect. As shown in Figure 2B, our data show that cell growth was decreased obviously in miR-150 inhibitors transfected MCF-7 (∼82.1%) or MDA-MB-231 (∼85.8%) cells on day 5 compared with their matched controls. We next examined the effect of miR-150 on the anchorage-dependent clonogenic ability of the breast cancer cells. As shown in Figure 2C and Figure S2A, we observed that the clonogenic ability was significantly increased in miR-150 mimics transfected MCF-7 and MDA-MB-231 cells, respectively, as compared with their respective controls. Down-regulation of miR-150 by antagomirs inhibited both the colony formation numbers and sizes. The clonogenic ability was decreased by 67% and 63% in miR-150 inhibitors transfected MCF-7 and MDA-MB-231 cells, respectively, as compared with their respective controls (Figure 2C, Figure S2A).

Finally, the proliferation of breast tumor cells was measured by their ability to incorporate BrdU after transfected with miR-150 mimics. MCF-7 or MDA-MB-231 cells transfected with miR-150, instead of miR-150 mimics negative control (NC) or mock transfection, showed a substantial (33% or 35%) increase in BrdU staining (Figure 2D, Figure S2B; P<0.05). Transfecting MCF-7 and MDA-MB-231 cells with miR-150 inhibitors, significantly reduced their sensitivity to the growth effect (Figure 2D, Figure S2B; P<0.05). To further investigate whether the miR-150 antagonism inhibits breast cancer development by inducing apoptosis, we used TUNEL-TMR staining to detect DNA fragmentation during programmed cell death. Transfection with miR-150 inhibitors, increased the percentage of TUNEL-positive cells by 6-fold in MCF-7 cells, and by 8-fold in MDA-MB-231cells (Figure 2E, Figure S2C; P<0.05 or P<0.01), but not in cells transfected with miR-150 inhibitor NC, suggesting that miR-150 may inhibit cancer cell death, whereas blockade of miR-150 leads to cell apoptosis.

These data provided evidence that ectopic miR-150 expression promotes breast cancer cell proliferation and growth.

### miR-150 promotes human breast cancer growth by targeting the pro-apoptotic purinergic P2X_7_ receptor

**Figure 3:**
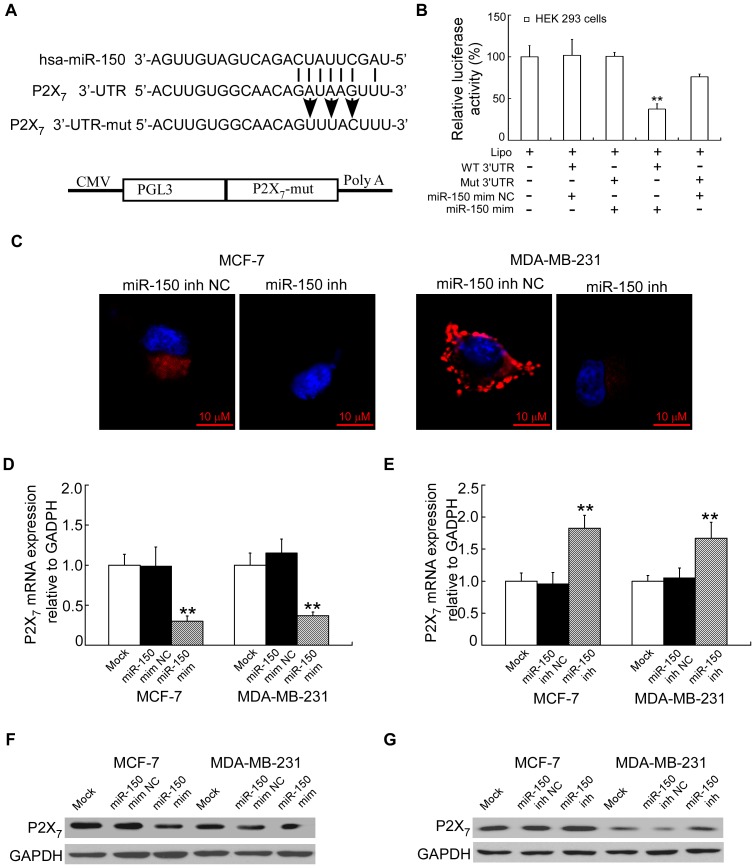
MiR-150 target the pro-apoptotic purinergic P2X_7_ receptor. (A) Sequence alignment of human miR-150 with 3′UTR of P2X_7_, miR-150 seed sequences matches in the 3′UTR regions of P2X_7_. (B) Relative luciferase activity was analyzed after the above reporter plasmids or control reporter plasmid were co-transfected with miR-150 mimics or negative control mimics in HEK 293 cells. ** p<0.01, One-way ANOVA with Bonferroni's multiple comparison t-test, compared to control reporter plasmids or Lipofectamine 2000 (Lipo). (C) FISH staining analysis for the expression of miR-150 in MCF-7 or MDA-MB-231cells treated with miR-150 inhibitors. (D–E) Expression of P2X_7_ mRNA in MCF-7 or MDA-MB-231 cells treated with miR-150 mimics, miR-150 inhibitors or appropriate NC. ** p<0.01, One-way ANOVA with Bonferroni's multiple comparison t-test, compared to matched NC or Mock. (F–G) Western blot analysis for the expression of P2X_7_ receptor in MCF-7 or MDA-MB-231 cells treated with miR-150 mimics, miR-150 inhibitors or matched NC. The in vitro data were depicted as mean ± SD of three independent experiments performed in triplicate.

Previous studies have shown the pro-apoptotic gene P2X_7_ is targeted by miR-150 instably in HeLa cells or E10 cells [8], [25]. Mechanisms that induce reduced expression of P2X_7_ receptor in cancer epithelial cells involve hypermethylation of the P2X_7_ gene and decreased transcription; enhanced degradation of the P2X_7_ transcript occurs through the action of microRNAs miR-186 and miR-150 [8], [11], [24]. We focused our attention on the P2X_7_ gene. For this, HEK 293 cells were transiently transfected with miR-150 mimics or miR-150 NC (non-targeted mimics) and a firefly luciferase reporter plasmid containing a region of P2X_7_ 3′UTR harboring miR-150 target site (Figure 3A). As a control, we also generated P2X_7_ 3′UTR mutant in the miR-150 target region to disrupt its binding site, which was used in co-transfection of breast cancer cells with miR-150 or miR-150 NC. Luciferase activity was measured after 24 h of transfection (Figure 3B). Our data demonstrated that relative luciferase unit (RLU) was decreased (>70%) in Wild type (WT) 3′UTR-P2X_7_ transfected HEK 293 cells that were co-transfected with miR-150 mimics compared to that co-transfected with miR-150 mimics NC. In addition, the expression of miR-150 in MCF-7 and MDA-MB-231 cells transfected with miR-150 inhibitors was identified by FISH staining (Figure 3C). The levels of P2X_7_ expression in MCF-7 and MDA-MB-231 cells by transfected with miR-150 were much lower than that in the control cells (Figure 3D–3F). In addition, transfection with miR-150 inhibitors, but not with the irrelevant miR-150 inhibitor NC, dramatically increased P2X_7_ expression, suggesting that P2X_7_ silencing in breast cancer cells is possibly mediated by miR-150 (Figure 3E–3G, Figure S3A–S3B).

Thus, our data strongly suggested that miR-150 negatively regulates the expression of P2X_7_ by directly targeting the 3′UTR of P2X_7_ transcript.

### miR-150 inhibitors reduces tumorigenesis of breast cancer xenografts

**Figure 4:**
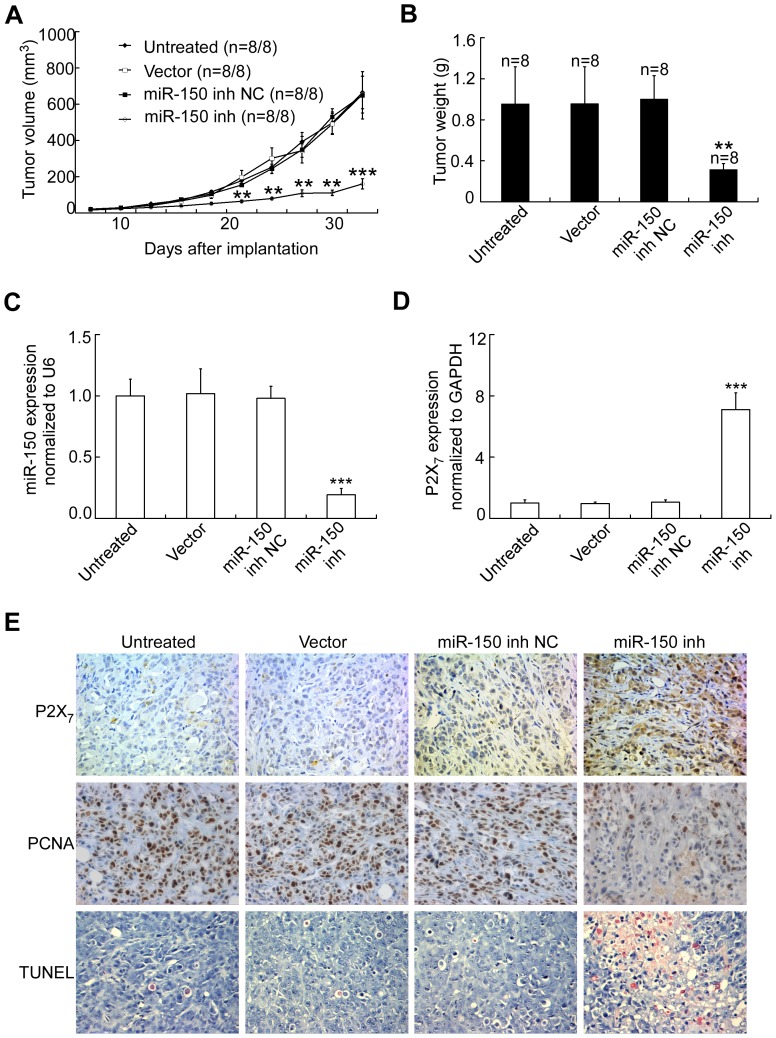
Reduction in miR-150 suppresses tumor growth in MDA-MB-231 cells xenografts implanted in BALB/c-nu mice. (A) Tumor volume of mice inoculated with MDA-MB-231 cells that stably expressing miR-150 inhibitor were measured up to 5 weeks. **P<0.01; ***P<0.001, One-way ANOVA compared to untreated, vector or NC groups. (B) Mean ± SD tumor weight of tumor-bearing mice upon necropsy (n = 8/group). **P<0.01, One-way ANOVA. (C) Expression of miR-150 normalized to U6, in the tumors of tumor-bearing mice, was determined by qRT-PCR. ***P<0.001, One-way ANOVA, comparing miR-150 inhibitors to untreated, vector or NC groups. (D) Expression of P2X_7_ mRNA relative to GAPDH mRNA, in the tumors of tumor-bearing mice, determined by qRT-PCR. ***P<0.001, One-way ANOVA, comparing miR-150 inhibitor to untreated, vector or NC groups. (E) Immunohistochemical staining (×400) for P2X_7_ receptor, PCNA or TUNEL.

As the miR-150 inhibitors reduces breast cancer cell growth *in vitro*, we further assessed its effect on tumor growth *in vivo*. A breast xenograft tumor model was established by mammary fat pad injection of MDA-MB-231 cells stably expressed miR-150 inhibitors or miR-150 inhibitor NC into female BALB/c-nu mice. Tumor volume and weight were measured twice a week. As shown in Figure 4A and 4B, reduction of miR-150 induced (Figure 4A–4B, Figure S4A) a significant inhibition in tumor growth, compared with mice treated with miR-150 NC inhibitor or vector alone. The expression level of miR-150 in MDA-MB-231 xenografts infected with miR-150 inhibitors was much lower than that in MDA-MB-231 xenografts transfected with miR-150 NC (Figure 4C). In line with miR-150 down-regulation, the descreased expression of miR-150 dramatically enhanced the expression of P2X_7_ mRNA in the breast cancer xenografts (Figure 4D). Immunohistochemistry with the anti-P2X_7_ antibody further confirmed that miR-150 inhibitors increased the expression of P2X_7_ in cancer cells of MDA-MB-231 xenografts (Figure 4E, Figure S4B). These data indicated that miR-150 inhibitors can efficiently reduce tumor growth *in vivo* via down-regulation of miR-150 and up-regulation of P2X_7_.

Although the tissue structure and cell morphology of MDA-MB-231 xenografts treated with miR-150 inhibitors were not different from those treated with miR-150 inhibitor NC or vector alone, transfection with miR-150 inhibitors significantly reduced the percentage of tumor cells expressing proliferating cell-associated antigen (PCNA) (Figure 4E, Figure S4C). In line with a significant decrease in PCNA, reduction expression of miR-150 significantly increased TUNEL-positive cells (apoptotic cells) in breast cancer xenografts (Figure 4E, Figure S4D). These data suggested that miR-150 inhibitors retarded tumor growth partly by inhibiting the proliferation of cancer cells. Thus, our findings suggested that blockade of miR-150 retards breast cancer development *in vivo*, probably by inhibiting proliferation and inducing apoptosis of cancer cells via P2X_7_ receptor up-regulation.

## Discussion

MicroRNAs can modulate a wide variety of biological processes [26]. Various miRNAs have been demonstrated to play specific roles in cancer cell differentiation, survival, tumor progression, and metastasis [27]. Numerous miRNAs have been reported to be differentially expressed in breast cancer cells, suggesting their involvement in breast cancer pathogenesis [28], [29]. However, the roles played by miRNAs in the pathogenesis of these diseases remained largely unknown. Since the role of miR-150 as a tumor suppressor or as an oncogene of tumor cell growth and metastasis in various cancers has been extensively studied [6], [7], [9], [30], we focused on its potential effectiveness in breast cancer. In this study, we found miR-150 was over-expressed in breast cancer cell lines and tissues. In the *in vitro* study, miR-150 promoted growth and proliferation of breast cancer cell lines, which was partially mediated by retrieving P2X_7_ expression. In the *in vivo* study, transfection of miR-150 inhibitors into MDA-MB-231 xenografts implanted subcutaneously in nude mice suppressed tumor growth, which was related to P2X_7_ up-regulation, reduced proliferation, and increased apoptosis of xenograft tumor cells.

Recent studies have indicated that abrogation of miR-150 markedly increased CXCR4 protein expression and enhanced BM-derived mononuclear cells mobilization and migration [9]. miR-150 over-expression in human skin BJ cells decreased caspase-3 activity, indicating an anti-apoptotic effect [31]. The transcription factor Myb is regulated by miR-150 post-transcriptionally and modulates cell fates in megakaryocyte-erythrocyte progenitors (MEP) [32], B-cell differentiation [33] and embryonic development [34]. miR-150 has also been identified as the best hit in several medium-scale profiling experiments designed to detect miRNAs dysregulated in tumors, including cancers of the lung [5], stomach [6], pancreas [7], and malignant lymphoma [35]. These differences may indicate that dysregulation of miRNAs in cancers depends on the cellular microenvironment. Therefore, miR-150 seems to be a useful treatment target for various kinds of malignant tumors.

In this study, we reported that the expression levels of miR-150 and its target proteins P2X_7_ in breast cancer cell lines as well as tumors and normal tissues of breast-cancer patients. Our investigation revealed that the expression level of miR-150 in malignant tissues was significantly higher when compared to their normal counterparts. Furthermore, we found a reverse-correlation in the expression of miR-150 and its target protein P2X_7_ receptor in examined malignant tissues. These molecular events led to enhanced proliferation and growth of breast cancer cells *in vitro* and *in vivo*. The results strongly support that over-expression of miR-150 enhances the levels of cancer cell survival factors and promotes cancer cell growth [6].

The development of cancer involves the alteration in expression levels of multiple genes. Therefore, a single protein product of an oncogene may not accurately reflect the status of the disease. However, a miRNA may regulate multiple coding genes that are related to tumor growth, and thus, is more likely to predict disease outcome more precisely and effectively. Apoptosis is a regulated homeostatic process orchestrated by the host's genome of selective cell deletion without stimulating inflammatory response. Dysregulation of apoptotic cell death has been implicated in status of disease and in the neoplastic transformation. Among the pro-apoptotic systems that operate in epithelial tissues, the P2X_7_ mechanism is probably the most important because the P2X_7_ receptor is expressed mainly by proliferating epithelial cells [36], thereby directly controlling the growth of epithelia. P2X_7_ receptor is an ATP-gated cation channel leading to Ca^2+^ release and to pleiotropic effects [11]. P2X_7_-mediated apoptosis plays a role in cell differentiation and aging [37]. Low levels of P2X_7_ receptor have been linked to cancer development. Reduced cellular P2X_7_ receptor content has been proposed as a biomarker for human breast, bladder, ectocervix, endocervix, and endometrial cancers [15]. Mechanisms that induced reduced expression of P2X_7_ receptor in cancer epithelial cells involved hypermethylation of the P2X_7_ gene and decreased transcription; enhanced degradation of the P2X_7_ transcript occurs through the action of miR-186 and miR-150 [8], [10], [16]. The 3′UTR of the human P2X_7_ contains sequences that confer instability to the P2X_7_ transcript. The human P2X_7_ 3′UTR contains binding sites for miR-186 and miR-150 that confer instability to the P2X_7_ transcript. Over-expression of miR-186 and miR-150 inhibits the synthesis of P2X_7_ mRNA, while inhibition of miR-186 and miR-150 up-regulates the synthesis of P2X_7_ mRNA and increases ligand-induced P2X_7_ pro-apoptotic effects [8]. When we transfected double-stranded miR-150 mimics oligonucleotides (miR-150 mimics) and the full-length 3′UTR-P2X_7_ luciferase reporter into HEK 293 cells, which lack endogenous expression of the P2X_7_ receptor, we found that miR-150 inhibitors increased luciferase activity, whereas miR-150 mimics decreased luciferase activity. The level of P2X_7_ expression in MDA-MB-231 and MCF-7 cells transfected with miR-150 was much lower than in the control cells. In addition, transfection with miR-150 inhibitors, but not with the irrelevant miR-NC inhibitor, dramatically increased P2X_7_ expression, suggesting that P2X_7_ silencing in breast cancer cells is possibly mediated by miR-150. Our results are in accordance with previous results reported by Zhou *et al.* [8] that post-transcriptional regulation of P2X_7_ mRNA expression involves miR-150. These data suggest that miR-150 stimulates a decrease in P2X_7_ mRNA steady-state levels by targeting instability sites within the 3′UTR of the P2X_7_ gene. Our results also reveal that P2X_7_ receptor expression is already decreased in the early phases of the breast neoplasia; this could abrogate apoptosis and lead to unstable tissue kinetics, favoring an increase in total cell number and tumor cellular expansion in cells exposed to the carcinogenic stimulus. The data suggest that the reduced expression of P2X_7_ receptor in cancer epithelial cell is the result of high steady-state levels of miR-150 in cancer cells, which activate the instability domains and decrease P2X_7_ mRNA levels possibly by inducing degradation of the transcript.

Finally, our findings in MDA-MB-231 xenografts demonstrated that inhibition of miR-150 reversed growth features of breast cancer cells and induced them to apoptosis. Decrease of miR-150 retarded the progression of breast cancer xenografts. Therefore, inhibition of miR-150 may provide novel therapeutic strategy against breast cancers. In contrast to artificially synthetic small interfering RNA (siRNA), miRNAs are endogenous molecules existing in normal cells, which may minimize their unexpected off-target silencing effects. Because a miRNA molecule targets to a set of coding genes rather than a single one, therapies based on miRNA interference could be more potent in cancer treatment by targeting multiple molecular pathways. Moreover, miRNA inhibitors or mimics could potentially be used as single therapeutic agents or in combination with other conventional chemotherapies/radiotherapies [38], [39] to achieve an optimal therapeutic effect.

In conclusion, our study suggests that miR-150 is an anti-apoptotic factor in breast cancer that maintains tumor cell growth, and thus, may play an important role for the development of malignancy. The study sheds new light on the specific function of miR-150 and its mechanism in breast cancer proliferation, and suggests that targeting miR-150 may provide a potential therapeutic strategy for blocking proliferation in breast cancer.

## Methods

### Tissues from breast cancer patients

Breast tumor samples and adjacent normal tissues were obtained at biopsy from 80 breast-cancer patients (Table S1) in the breast tumor center, Sun Yat-Sen Memorial Hospital, Sun Yat-sen University, from January 2008 to December 2011. All the patients recruited into the present study did not receive radiotherapy or chemotherapy or any other treatment before and after operation. Surgical specimens of the tumor resection were collected, and lumps of tumors as well as adjacent normal tissues, which were at least 2 cm distal to tumor margins, were snap-frozen in liquid nitrogen for miRNA assay. In addition, the remaining tissues were embedded for studies of histology, immunohistochemistry, and *in situ* hybridization. All patients signed informed consent approving the use of their tissues for research purposes and the study was approved by the Ethics Committee of Sun Yat-Sen Memorial Hospital (Ethical number: 2010-09).

### Cell culture and transfection

The following is the sources of cells and reagents used. Human embryonic kidney-293 cells (HEK 293 cells), the breast cancer cell lines (MDA-MB-231, MCF-7), and human mammary epithelial cell line (MCF-10A) were obtained from the ATCC and maintained in RPMI 1640 or Dulbecco's Modified Eagle Medium (DMEM) with 10% fetal bovine serum (FBS) and 1% antibiotics (Invitrogen, USA). Transfection of the cells with miRNA mimics or miRNA inhibitors (Genepharma, China) was performed using Lipofectamine 2000 (Invitrogen, USA) as previously described [40].

### Quantitative reverse transcription-PCR (qRT-PCR)

Total RNA was extracted from cells or tissues using Ezol according to the manufacturer's instructions (GenePharma, China). Real-time PCR was carried out using FTC3000 (Funglyn, Canada). The primers used for miR-150, U6 snRNA, P2X_7_ and GAPDH are as follows [8]: miR-150 forward, 5′-CAGTATTCTCTCCCAACCCTTGTA-3′; miR-150 reverse, 5′-AATGGATGATCTCGTCAGTCTGTT-3′, U6 snRNA forward, 5′-ATTGGAACGATACAGAGAAGATT-3′; U6 snRNA reverse, 5′-GGAACGCTTCACGAATTTG-3′, P2X_7_ forward, 5′-CATGAGAAGTATGACAACAGCCT-3′; P2X_7_ reverse, 5′-AGTCCTTCCACGATACCAAAGT-3′, GAPDH forward, 5′-GCTGCTTAGAAAGGAGGCG-3′; GAPDH, 5′-GGGAGTTGAGATGGGAGGC-3′. PCR amplification consisted of an initial denaturation step at 95°C for 3 min, followed by 40 cycles of 95°C for 15 s, 62°C for 30 s, and 72°C for 30 s. All primers and qRT-PCR quantitation Kit were purchased from GenePharma (GenePharma, China). Standard curves were generated, and the relative amount of miR-150 or P2X_7_ was normalized to the amount of U6 snRNA or GAPDH, respectively.

### MicroRNA *in situ* hybridization

miR-150 expression was examined by *in situ* hybridization [41] on the formalin-fixed and paraffin-embedded sections of breast cancers. This assay was performed according to the manufacturer's protocol (Exiqon, Denmark). Briefly, after demasking, microRNA was hybridized to 5′-DIG-labeled LNA probes. Then the digoxigenins were recognized by a specific anti-DIG antibody (Abcam, USA) directly conjugated to alkaline phosphatase. The nuclei were counterstained with fast red. A total of 1000 tumor cells were counted randomly in each section.

### Fluorescent in situ hybridization (FISH)

FISH hybridization was performed in breast cancer cells under the following conditions: Cells were prehybridized at 45°C for 2 h and hybridized at 45°C for 16 h. After the hybridization, cells were washed with 2 x SSC at 45°C. Then, probe detection was performed using TRITC-anti-DIG antibody (Roche, Switzerland) and DAPI was used as counterstain. The coverslips were evaluated using a Zeiss LSM 710 confocal microscope (Zeiss, Germany).

### Immunofluorescence staining

Cells were stained for immunofluorescence on coverslips. After fixation and permeabilization, the cells were incubated with primary antibody against P2X_7_ (Santa Cruz, USA) and then incubated with rhodamine-conjugated secondary antibody (Invitrogen, USA). The coverslips were counterstained with DAPI and imaged under a Zeiss LSM 710 confocal microscope (Zeiss, Germany).

### Immunohistochemistry

P2X_7_ receptor expression was examined by immunohistochemistry on paraffin-embedded tissue sections. Briefly, rabbit anti-P2X_7_ polyclonal antibody (Santa Cruz, USA) was used as the primary antibody for overnight incubation at 4°C. The sections were then treated with secondary antibody, followed by further incubation with streptavidin-horseradish peroxidase complex. Diaminobenzidine (Dako, USA) was used as a chromogen and the nuclei were counterstained with hematoxylin. The percentage of positively staining tumor cells was calculated per field of view, with at least 20 view fields per section evaluated at ×400 magnification.

### Luciferase assay

To evaluate the function of miR-150, the 3′UTR of P2X_7_ with a miR-150 targeting sequence was cloned into a pGL3-promoter luciferase reporter vector (Ibsbio, China). Luciferase assays were carried out in HEK 293 cells. To correct transfection efficiency, a luciferase reporter vector without the miR-150 target was transfected in parallel. Luciferase activities were assayed using a luciferase assay kit (Promega, USA), and target effect was expressed as relative luciferase activity of the reporter vector with target sequence over the one without target sequence. Each assay was performed three times.

### Cell proliferation assay and clonogenicity assays

The effect of miR-150 on proliferation of breast cancer cells was evaluated by the MTT assay. MDA-MB-231 and MCF-7 cells were plated in 96-well culture plates (3×10^3^ per well). After 24h incubation, the cells were treated with miR-150 inhibitors, anti-miR-NC (miR-NC inhibitor), miR-150 mimics, miR-150-NC mimics and mock for 48 hours. MTT (0.5 mg/ml; Sigma-Aldrich, USA) was then added to each well (200 µl/well). After 4 hours of additional incubation, MTT solution was discarded and 200 µl of DMSO (Sigma, USA) was added and the plates shaken gently. The absorbance was measured on an ELISA reader at a wavelength of 570 nm. For clonogenicity assays, cells were cultured and subsequently transfected with microRNAs mimics (miR-150 or miR-NC). Following 48 hours transfection, cells were trypsinized and plated in 6-well plates at a density of 1×10^3^ cells/well in regular media for colony formation. After two weeks, colonies were fixed with methanol, stained with crystal violet, photographed and counted. Each experiment was performed in triplicate.

### TUNEL assay

Terminal deoxynucleotidyl transferase–mediated dUTP labeling (TUNEL) assay was done using an *in situ* apoptosis detection kit (R&D Systems, USA). Briefly, after digesting with Protease K, TdT reaction mix was applied to the cells for incubation at 37°C for 60 min, followed by incubation with streptavidin horseradish peroxidase for 10 min. The final reaction of the product was visualized by 3,3′-diaminobenzidine. Approximately 1,000 tumor cells were counted in each section, and apoptotic index was expressed as the percentage of TUNEL-positive tumor cells.

### Western blotting

Protein extracts were resolved through 10% SDS-polyacrylamide gel electrophoresis, transferred to polyvinylidene difluoride membranes (BioRad, USA), probed with antibody against P2X_7_ receptor (Santa Cruz, USA), and GAPDH from Santa Cruz (Santa Cruz, USA), and then with peroxidase-conjugated secondary antibody (ProteinTech Group, USA), followed visualization using chemiluminescence (GE, USA).

### Animal experiments

All animal procedures were approved by the Animal Care and Use Committee of Sun Yat-Sen University (Protocol number: 2010-09) and conformed to the legal mandates and national guidelines for the care and maintenance of laboratory animals. MDA-MB-231 breast cancer cells (1×10^6^/mouse) (untransduced or transduced with miRNA-expressing vector LV4-miR-150 inhibitor-GFP-Luc or LV4-NC-GFP-Luc were injected subcutaneously into the mammary fat pad of 5-week-old BALB/c-nu mice. After tumors were detected, tumor size was measured and calculated as volume (mm^3^) = length×width^2^ ×0.5 for up to 5 weeks. Then tumor xenografts were harvested, weighed, and processed for histology. Cryosections (4 µm) were used for TUNEL assays as well as immunohistochemistry for P2X_7_ receptor (Santa Cruz, USA). The miR-150 level of tumors was also determined by using qRT-PCR. Animal experiments were performed in accordance with the institutional guidelines of the university committee on the use and care of animals.

### Statistical analysis

All statistical analyses were carried out using SPSS for Windows version 16.0 (SPSS, Chicago, IL, USA). The differences between the means were tested by an independent sample t-test, one-way analysis of variance (ANOVA) or Bonferroni's multiple comparison t-test. The chi-squared test was used to compare the the clinicopathological status and the expression of miR-150 or P2X_7_. Each experiment was performed at least three times, independently. Measurement data were presented as mean ± standard deviation (SD). *P*<0.05 was considered statistically significant. Corrected P-value =  p-value * n<0.05

## Supporting Information

Figure S1 **The level of miR-150 correlates inversely with P2X_7_ in breast cancer cell lines.** Western-blotting analysis for the expression of P2X_7_ receptor in breast cancer cell lines. The bands of P2X_7_ were densitometrically evaluated. Data are shown in arbitrary units (AU) normalized to MCF-10A cells as the mean ± SD of three independent experiments. **; p<0.01; ***; p<0.001, Student's t-test for MCF-7 or MDA-MB-231 cells compared to MCF-10A cells. ##; p<0.01MDA-MB-231 cells compared to MCF-7 cells.
(TIF)

Figure S2 **miR-150 promotes the cell growth and inhibits the cell apoptosis of breast cancer cells.** (A) Bars represent the mean of total number of colonies ± SD of three independent experiments. (B) Percentage of BrdU^+^ cells determined by flow cytometric analysis of BrdU immunostaining in MCF-7 or MDA-MB-231 cells transfected with miR-150 mimics or miR-150 inhibitors. (C) Cell apoptosis assay performed in MCF-7 or MDA-MB-231 cells 2 days after miR-150 inhibitor transfection, Data are the mean of three determinations that gave similar results. * p<0.05; ** p<0.01; *** p<0.001, One-way ANOVA compared to matched NC or Mock.
(TIF)

Figure S3 **miR-150 target the pro-apoptotic purinergic P2X_7_ receptor.** (A-B) Western blot analysis for the expression of P2X_7_ receptor in MCF-7 and MDA-MB-231 cells treated with miR-150 mimics or miR-150 inhibitors. Data are shown in arbitrary units (AU) normalized to Mock as the mean ± SD of three independent experiments. ** p<0.01, One-way ANOVA compared to matched NC or Mock. ##P<0.01, One-way ANOVA compared to matched NC or Mock.
(TIF)

Figure S4 **Reduction in miR-150 suppresses tumor growth in MDA-MB-231 cells xenografts implanted in BALB/c-nu mice.** (A) Representative photographs of the tumors of each group from two independent experiments. (B) Quantification of P2X_7_ (B), PCNA (C) or TUNEL (D) in MDA-MB-231 derived tumor sections is shown. * P<0.05; ** P<0.01; *** P<0.001, One-way ANOVA compared to inhibitor NC, vector or untreated mice.
(TIF)

Table S1 **Correlation among clinicopathological status and the expression of miR-150 or P2X7 in breast cancer patients.** Note: *, grading in 80 cases of invasive ductal carcinoma; **, Chi-squared test.
(DOC)

## References

[1] Veronesi U, Boyle P, Goldhirsch A, Orecchia R, Viale G (2005) Breast cancer. Lancet 365: 1727–1741. 15894099 10.1016/S0140-6736(05)66546-4

[2] He L, Hannon GJ (2004) MicroRNAs: small RNAs with a big role in gene regulation. Nat Rev Genet 5: 522–531. 15211354 10.1038/nrg1379

[3] Vasilescu C, Rossi S, Shimizu M, Tudor S, Veronese A, et al (2009) MicroRNA fingerprints identify miR-150 as a plasma prognostic marker in patients with sepsis. PLoS One 4: e7405. 19823581 10.1371/journal.pone.0007405 PMC2756627

[4] Zhou B, Wang S, Mayr C, Bartel DP, Lodish HF (2007) miR-150, a microRNA expressed in mature B and T cells, blocks early B cell development when expressed prematurely. Proc Natl Acad Sci U S A 104: 7080–7085. 17438277 10.1073/pnas.0702409104 PMC1855395

[5] Wang PY, Li YJ, Zhang S, Li ZL, Yue Z, et al (2010) Regulating A549 cells growth by ASO inhibiting miRNA expression. Mol Cell Biochem 339: 163–171. 20049626 10.1007/s11010-009-0380-2

[6] Wu Q, Jin H, Yang Z, Luo G, Lu Y, et al (2010) MiR-150 promotes gastric cancer proliferation by negatively regulating the pro-apoptotic gene EGR2. Biochem Biophys Res Commun 392: 340–345. 20067763 10.1016/j.bbrc.2009.12.182

[7] Srivastava SK, Bhardwaj A, Singh S, Arora S, Wang B, et al (2011) MicroRNA-150 directly targets MUC4 and suppresses growth and malignant behavior of pancreatic cancer cells. Carcinogenesis 32: 1832–1839. 21983127 10.1093/carcin/bgr223 PMC3220613

[8] Zhou L, Qi X, Potashkin JA, Abdul-Karim FW, Gorodeski GI (2008) MicroRNAs miR-186 and miR-150 down-regulate expression of the pro-apoptotic purinergic P2X7 receptor by activation of instability sites at the 3′-untranslated region of the gene that decrease steady-state levels of the transcript. J Biol Chem 283: 28274–28286. 18682393 10.1074/jbc.M802663200 PMC2568908

[9] Tano N, Kim HW, Ashraf M (2011) microRNA-150 regulates mobilization and migration of bone marrow-derived mononuclear cells by targeting Cxcr4. PLoS One 6: e23114. 22039399 10.1371/journal.pone.0023114 PMC3198444

[10] Gorodeski GI (2009) P2X7-mediated chemoprevention of epithelial cancers. Expert Opin Ther Targets 13: 1313–1332. 19845494 10.1517/14728220903277249

[11] Ortego M, Bustos C, Hernandez-Presa MA, Tunon J, Diaz C, et al (1999) Atorvastatin reduces NF-kappaB activation and chemokine expression in vascular smooth muscle cells and mononuclear cells. Atherosclerosis 147: 253–261. 10559511 10.1016/s0021-9150(99)00193-8

[12] Hughes JP, Hatcher JP, Chessell IP (2007) The role of P2X(7) in pain and inflammation. Purinergic Signal 3: 163–169. 18404430 10.1007/s11302-006-9031-1 PMC2096758

[13] Labrousse VF, Costes L, Aubert A, Darnaudery M, Ferreira G, et al (2009) Impaired interleukin-1beta and c-Fos expression in the hippocampus is associated with a spatial memory deficit in P2X(7) receptor-deficient mice. PLoS One 4: e6006. 19547756 10.1371/journal.pone.0006006 PMC2695542

[14] Eltom S, Stevenson CS, Rastrick J, Dale N, Raemdonck K, et al (2011) P2X7 receptor and caspase 1 activation are central to airway inflammation observed after exposure to tobacco smoke. PLoS One 6: e24097. 21915284 10.1371/journal.pone.0024097 PMC3167831

[15] Li X, Qi X, Zhou L, Fu W, Abdul-Karim FW, et al (2009) P2X(7) receptor expression is decreased in epithelial cancer cells of ectodermal, uro-genital sinus, and distal paramesonephric duct origin. Purinergic Signal 5: 351–368. 19399640 10.1007/s11302-009-9161-3 PMC2717318

[16] Zhou L, Luo L, Qi X, Li X, Gorodeski GI (2009) Regulation of P2X(7) gene transcription. Purinergic Signal 5: 409–426. 19609728 10.1007/s11302-009-9167-x PMC2717324

[17] Lenertz LY, Wang Z, Guadarrama A, Hill LM, Gavala ML, et al (2010) Mutation of putative N-linked glycosylation sites on the human nucleotide receptor P2X7 reveals a key residue important for receptor function. Biochemistry 49: 4611–4619. 20450227 10.1021/bi902083n PMC2895974

[18] Feng YH, Li X, Wang L, Zhou L, Gorodeski GI (2006) A truncated P2X7 receptor variant (P2X7-j) endogenously expressed in cervical cancer cells antagonizes the full-length P2X7 receptor through hetero-oligomerization. J Biol Chem 281: 17228–17237. 16624800 10.1074/jbc.M602999200 PMC2409001

[19] Barh D, Parida S, Parida B, Viswanathan G (2008) Let-7, miR-125, miR-205, and miR-296 are prospective therapeutic agents in breast cancer molecular medicine. Gene Ther Mol Biol 2008 12: 189–206.

[20] NC D'Amato, H Gu, M Lee, R Heinz, NS Spoelstra, et al. (2012) A functional role for miR-150 in breast cancer. Cancer Res 72 (24 Suppl): Abstract nr P5-10-06.

[21] Li X, Qi X, Zhou L, Catera D, Rote NS, et al (2007) Decreased expression of P2X7 in endometrial epithelial pre-cancerous and cancer cells. Gynecol Oncol 106: 233–243. 17482244 10.1016/j.ygyno.2007.03.032 PMC2398694

[22] Kujoth GC, Leeuwenburgh C, Prolla TA (2006) Mitochondrial DNA mutations and apoptosis in mammalian aging. Cancer Res 66: 7386–7389. 16885331 10.1158/0008-5472.CAN-05-4670

[23] Rodriguez-Nieto S, Zhivotovsky B (2006) Role of alterations in the apoptotic machinery in sensitivity of cancer cells to treatment. Curr Pharm Des 12: 4411–4425. 17168751 10.2174/138161206779010495

[24] Di Virgilio F, Ferrari D, Adinolfi E (2009) P2X(7): a growth-promoting receptor-implications for cancer. Purinergic Signal 5: 251–256. 19263244 10.1007/s11302-009-9145-3 PMC2686832

[25] Weng T, Mishra A, Guo Y, Wang Y, Su L, et al (2012) Regulation of lung surfactant secretion by microRNA-150. Biochem Biophys Res Commun 422: 586–589. 22595456 10.1016/j.bbrc.2012.05.030 PMC3377846

[26] Navarro A, Monzo M (2010) MicroRNAs in human embryonic and cancer stem cells. Yonsei Med J 51: 622–632. 20635434 10.3349/ymj.2010.51.5.622 PMC2908867

[27] Navarro A, Marrades RM, Vinolas N, Quera A, Agusti C, et al (2009) MicroRNAs expressed during lung cancer development are expressed in human pseudoglandular lung embryogenesis. Oncology 76: 162–169. 19209007 10.1159/000201569

[28] Ma L, Reinhardt F, Pan E, Soutschek J, Bhat B, et al (2010) Therapeutic silencing of miR-10b inhibits metastasis in a mouse mammary tumor model. Nat Biotechnol 28: 341–347. 20351690 10.1038/nbt.1618 PMC2852471

[29] Valastyan S, Reinhardt F, Benaich N, Calogrias D, Szasz AM, et al (2009) A pleiotropically acting microRNA, miR-31, inhibits breast cancer metastasis. Cell 137: 1032–1046. 19524507 10.1016/j.cell.2009.03.047 PMC2766609

[30] Wang M, Tan LP, Dijkstra MK, van Lom K, Robertus JL, et al (2008) miRNA analysis in B-cell chronic lymphocytic leukaemia: proliferation centres characterized by low miR-150 and high BIC/miR-155 expression. J Pathol 215: 13–20. 18348159 10.1002/path.2333

[31] Ovcharenko D, Kelnar K, Johnson C, Leng N, Brown D (2007) Genome-scale microRNA and small interfering RNA screens identify small RNA modulators of TRAIL-induced apoptosis pathway. Cancer Res 67: 10782–10788. 18006822 10.1158/0008-5472.CAN-07-1484

[32] Lu J, Guo S, Ebert BL, Zhang H, Peng X, et al (2008) MicroRNA-mediated control of cell fate in megakaryocyte-erythrocyte progenitors. Dev Cell 14: 843–853. 18539114 10.1016/j.devcel.2008.03.012 PMC2688789

[33] Xiao C, Calado DP, Galler G, Thai TH, Patterson HC, et al (2007) MiR-150 controls B cell differentiation by targeting the transcription factor c-Myb. Cell 131: 146–159. 17923094 10.1016/j.cell.2007.07.021

[34] Lin YC, Kuo MW, Yu J, Kuo HH, Lin RJ, et al (2008) c-Myb is an evolutionary conserved miR-150 target and miR-150/c-Myb interaction is important for embryonic development. Mol Biol Evol 25: 2189–2198. 18667440 10.1093/molbev/msn165

[35] Watanabe A, Tagawa H, Yamashita J, Teshima K, Nara M, et al (2011) The role of microRNA-150 as a tumor suppressor in malignant lymphoma. Leukemia 25: 1324–1334. 21502955 10.1038/leu.2011.81

[36] Li X, Zhou L, Feng YH, Abdul-Karim FW, Gorodeski GI (2006) The P2X7 receptor: a novel biomarker of uterine epithelial cancers. Cancer Epidemiol Biomarkers Prev 15: 1906–1913. 17035398 10.1158/1055-9965.EPI-06-0407 PMC2376759

[37] Soti C, Sreedhar AS, Csermely P (2003) Apoptosis, necrosis and cellular senescence: chaperone occupancy as a potential switch. Aging Cell 2: 39–45. 12882333 10.1046/j.1474-9728.2003.00031.x

[38] Krutzfeldt J, Rajewsky N, Braich R, Rajeev KG, Tuschl T, et al (2005) Silencing of microRNAs in vivo with ‘antagomirs’. Nature 438: 685–689. 16258535 10.1038/nature04303

[39] Jeyaseelan K, Herath WB, Armugam A (2007) MicroRNAs as therapeutic targets in human diseases. Expert Opin Ther Targets 11: 1119–1129. 17665982 10.1517/14728222.11.8.1119

[40] Gong C, Yao Y, Wang Y, Liu B, Wu W, et al (2011) Up-regulation of miR-21 mediates resistance to trastuzumab therapy for breast cancer. J Biol Chem 286: 19127–19137. 21471222 10.1074/jbc.M110.216887 PMC3099726

[41] Sun L, Yao Y, Liu B, Lin Z, Lin L, et al (2011) MiR-200b and miR-15b regulate chemotherapy-induced epithelial-mesenchymal transition in human tongue cancer cells by targeting BMI1. Oncogene 31: 432–445. 21725369 10.1038/onc.2011.263

