# Supplementary figures and images for: miR-150 Promotes Human Breast Cancer Growth and Malignant Behavior by Targeting the Pro-Apoptotic Purinergic P2X7 Receptor
Source: PLoS One. 2013 Dec 2;8(12):e80707. doi: 10.1371/journal.pone.0080707 (PMC3846619; doi:10.1371/journal.pone.0080707)

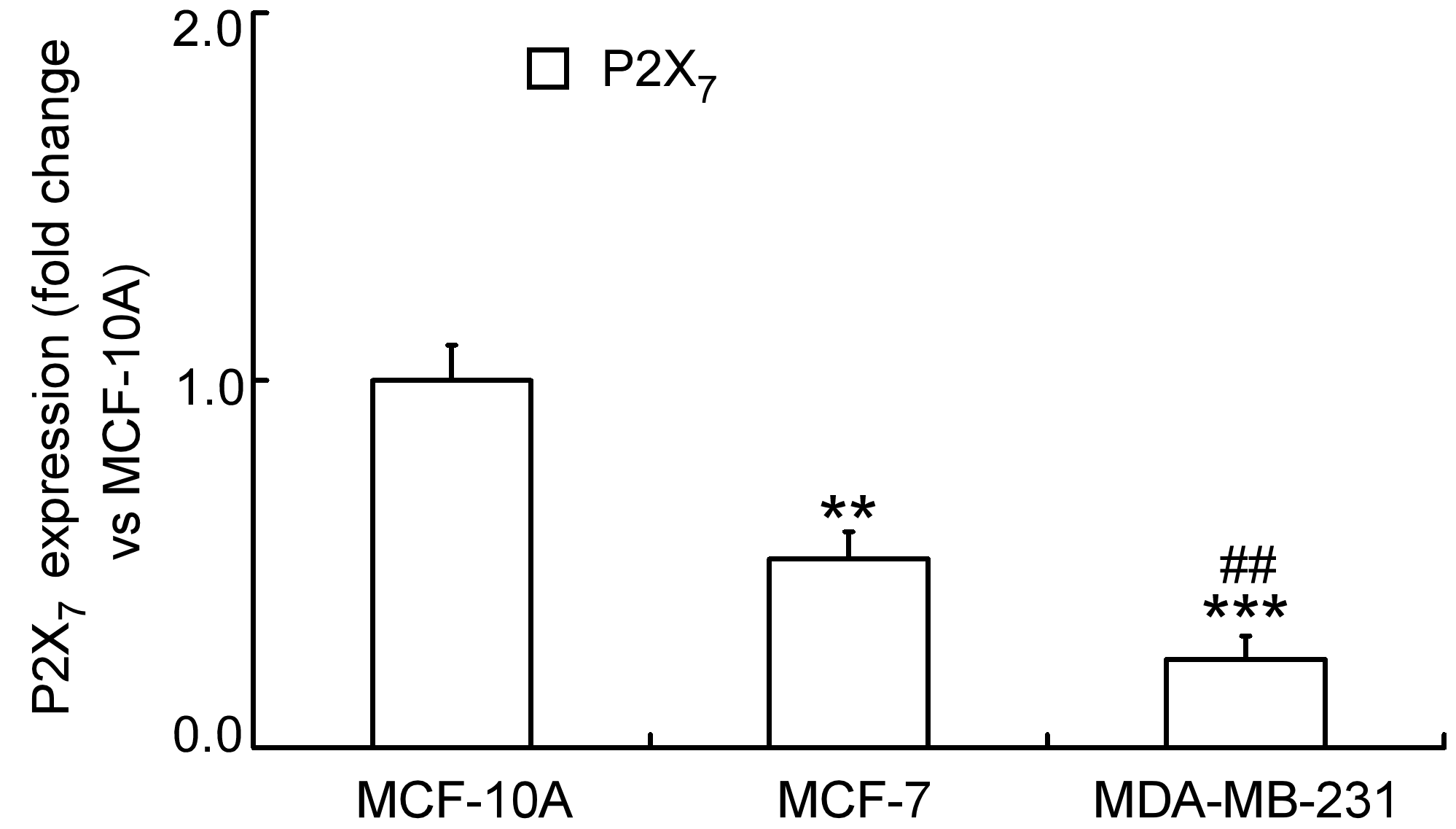

Supplement: Figure S1 — The level of miR-150 correlates inversely with P2X7 in breast cancer cell lines. Western-blotting analysis for the expression of P2X7 receptor in breast cancer cell lines. The bands of P2X7 were densitometrically evaluated. Data are shown in arbitrary units (AU) normalized to MCF-10A cells as the mean ± SD of three independent experiments. **; p<0.01; ***; p<0.001, Student's t-test for MCF-7 or MDA-MB-231 cells compared to MCF-10A cells. ##; p<0.01MDA-MB-231 cells compared to MCF-7 cells. (TIF) [file pone.0080707.s001.tif]

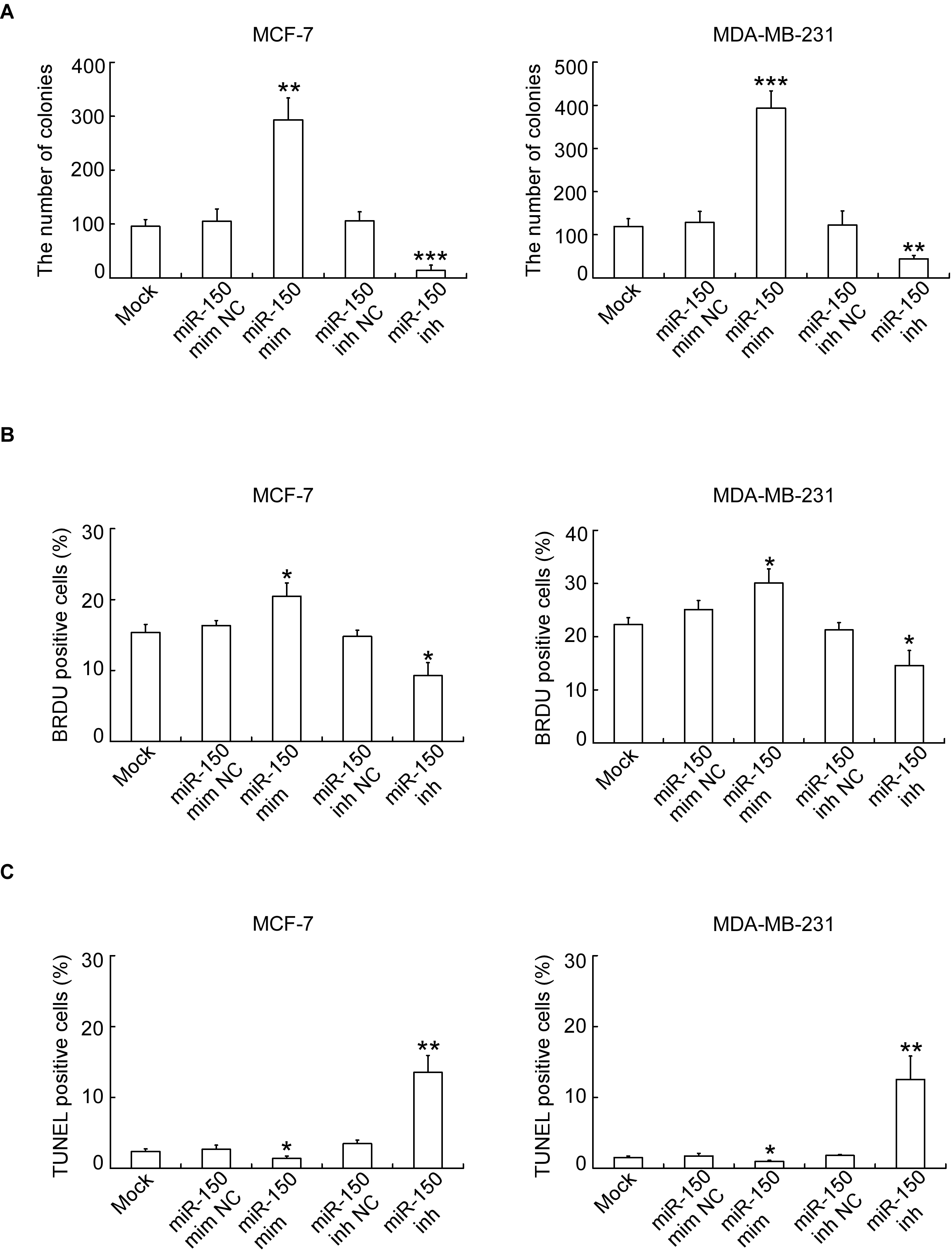

Supplement: Figure S2 — miR-150 promotes the cell growth and inhibits the cell apoptosis of breast cancer cells. (A) Bars represent the mean of total number of colonies ± SD of three independent experiments. (B) Percentage of BrdU+ cells determined by flow cytometric analysis of BrdU immunostaining in MCF-7 or MDA-MB-231 cells transfected with miR-150 mimics or miR-150 inhibitors. (C) Cell apoptosis assay performed in MCF-7 or MDA-MB-231 cells 2 days after miR-150 inhibitor transfection, Data are the mean of three determinations that gave similar results. * p<0.05; ** p<0.01; *** p<0.001, One-way ANOVA compared to matched NC or Mock. (TIF) [file pone.0080707.s002.tif]

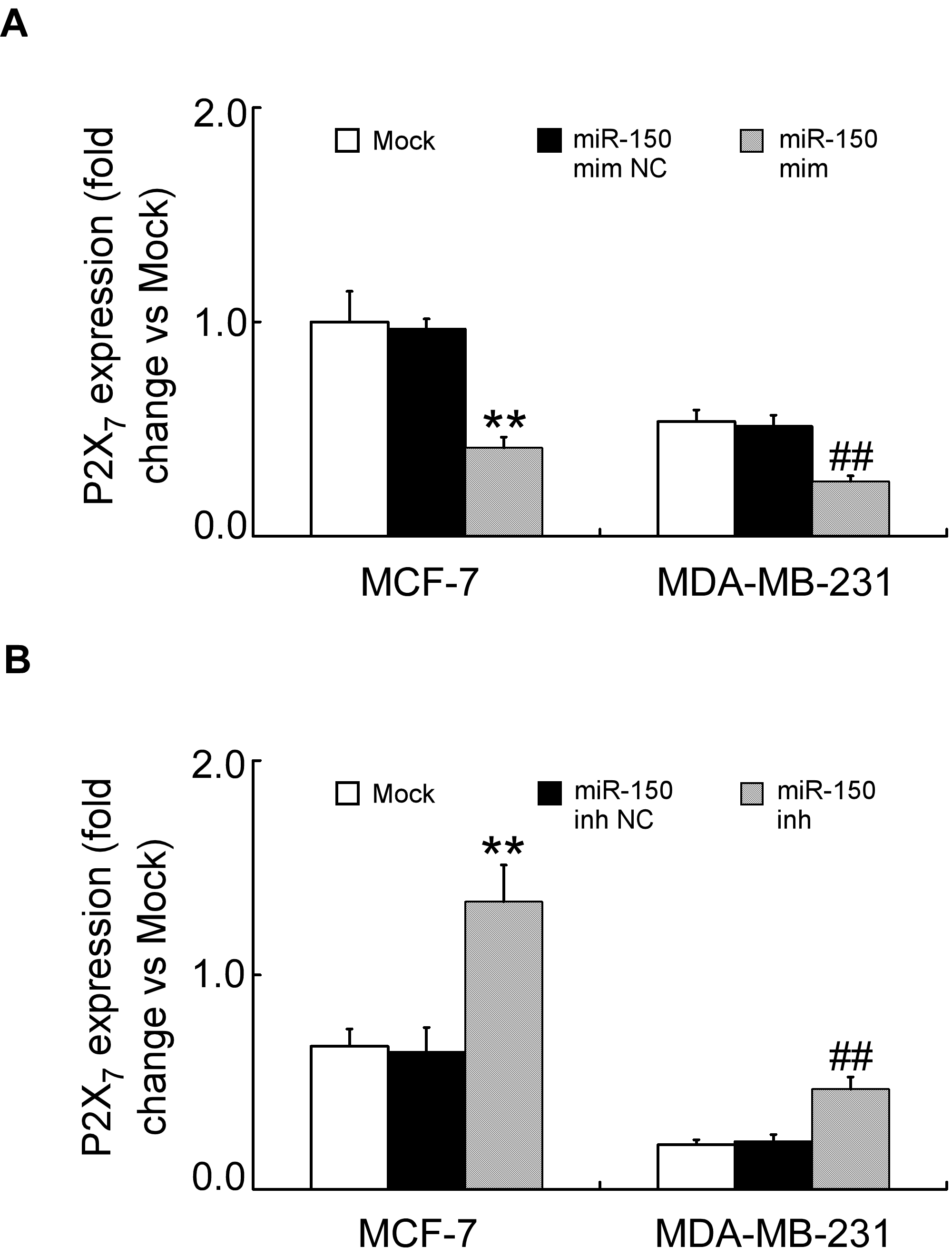

Supplement: Figure S3 — miR-150 target the pro-apoptotic purinergic P2X7 receptor. (A-B) Western blot analysis for the expression of P2X7 receptor in MCF-7 and MDA-MB-231 cells treated with miR-150 mimics or miR-150 inhibitors. Data are shown in arbitrary units (AU) normalized to Mock as the mean ± SD of three independent experiments. ** p<0.01, One-way ANOVA compared to matched NC or Mock. ##P<0.01, One-way ANOVA compared to matched NC or Mock. (TIF) [file pone.0080707.s003.tif]

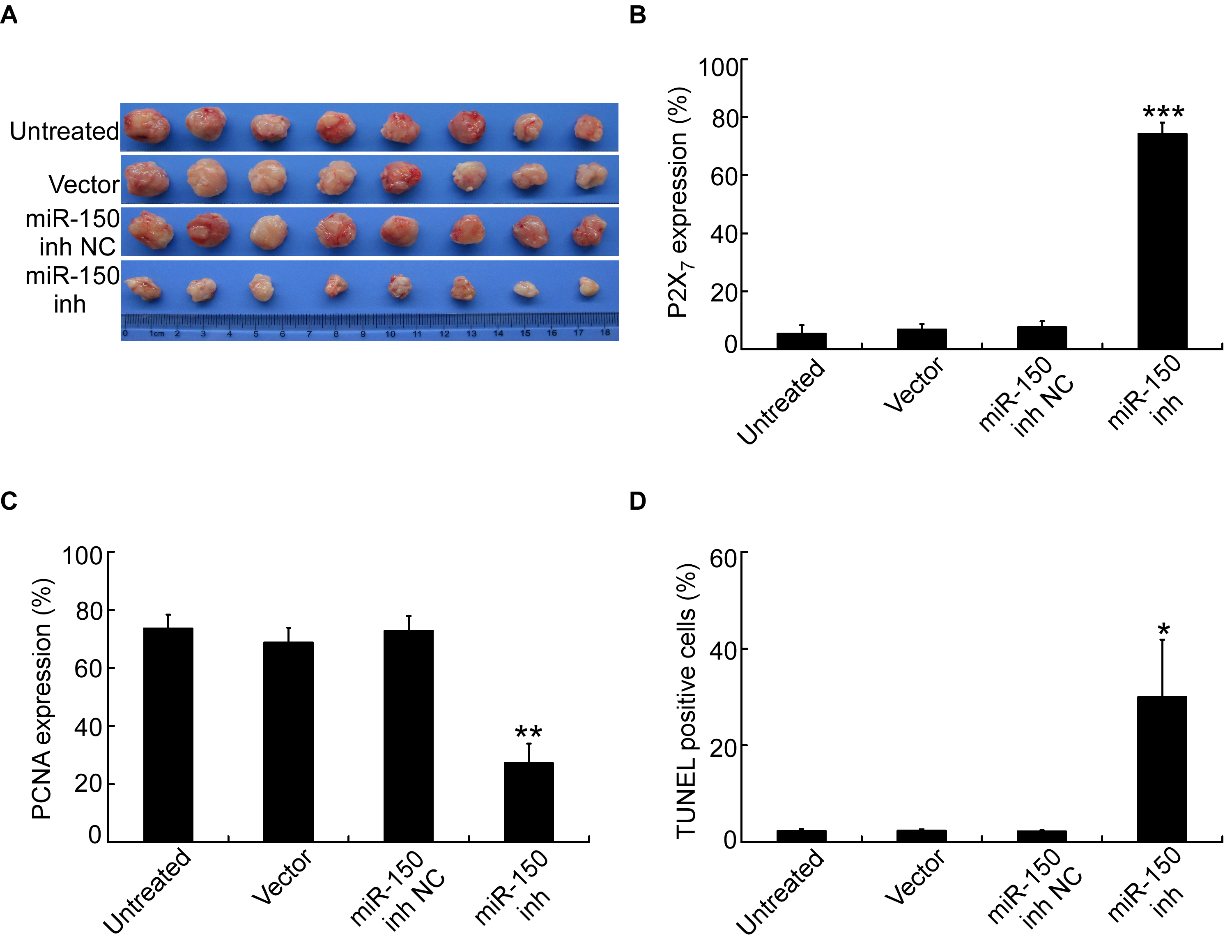

Supplement: Figure S4 — Reduction in miR-150 suppresses tumor growth in MDA-MB-231 cells xenografts implanted in BALB/c-nu mice. (A) Representative photographs of the tumors of each group from two independent experiments. (B) Quantification of P2X7 (B), PCNA (C) or TUNEL (D) in MDA-MB-231 derived tumor sections is shown. * P<0.05; ** P<0.01; *** P<0.001, One-way ANOVA compared to inhibitor NC, vector or untreated mice. (TIF) [file pone.0080707.s004.tif]
