# Supplementary material for: miR-150 Promotes Human Breast Cancer Growth and Malignant Behavior by Targeting the Pro-Apoptotic Purinergic P2X7 Receptor
Source: PLoS One. 2013 Dec 2;8(12):e80707. doi: 10.1371/journal.pone.0080707 (PMC3846619; doi:10.1371/journal.pone.0080707)
Supplement: Table S1 — Correlation among clinicopathological status and the expression of miR-150 or P2X7 in breast cancer patients. Note: *, grading in 80 cases of invasive ductal carcinoma; **, Chi-squared test. (DOC) [file pone.0080707.s005.doc]

Table S1 Correlation among clinicopathological status and the expression of miR-150, or P2X_7_ in breast cancer patients

| Characteristics | miR-150  No. of low No. of high expression expression | | *P* value** | P2X_7_  No. of low No. of high expression expression | | *P* value** |
| --- | --- | --- | --- | --- | --- | --- |
| Age  ≤45  >45  Tumor size (cm)  ≤2  >2  Histological grade*  Ⅰ  Ⅱ  Ⅲ | 20  26  19  27  8  23  15 | 13  21  9  25  3  8  23 | 0.638  0.169  0.008 | 16  23  15  24  4  9  26 | 17  24  13  28  7  22  12 | 0.968  0.527    0.003 |

Note:***,** grading in cases of invasive ductal carcinoma. **, Chi-squared test
